# Urbanization and the global malaria recession

**DOI:** 10.1186/1475-2875-12-133

**Published:** 2013-04-17

**Authors:** Andrew J Tatem, Peter W Gething, David L Smith, Simon I Hay

**Affiliations:** 1 Department of Geography and Environment, University of Southampton, Highfield, Southampton, UK; 2 Fogarty International Center, National Institutes of Health, Bethesda, MD 20892, USA; 3 Spatial Ecology and Epidemiology Group, Tinbergen Building, Department of Zoology, University of Oxford, South Parks Road, Oxford OX1 3PS, UK; 4 Department of Epidemiology, Johns Hopkins Bloomberg School of Public Health, Baltimore, MD, USA; 5 Malaria Research Institute, Johns Hopkins Bloomberg School of Public Health, Baltimore, MD, USA

**Keywords:** Urbanization, Global malaria endemicity, *Plasmodium falciparum*, *Plasmodium vivax*, Malaria elimination

## Abstract

**Background:**

The past century has seen a significant contraction in the global extent of malaria transmission, resulting in over 50 countries being declared malaria free, and many regions of currently endemic countries eliminating the disease. Moreover, substantial reductions in transmission have been seen since 1900 in those areas that remain endemic today. Recent work showed that this malaria recession was unlikely to have been driven by climatic factors, and that control measures likely played a significant role. It has long been considered, however, that economic development, and particularly urbanization, has also been a causal factor. The urbanization process results in profound socio-economic and landscape changes that reduce malaria transmission, but the magnitude and extent of these effects on global endemicity reductions are poorly understood.

**Methods:**

Global data at subnational spatial resolution on changes in malaria transmission intensity and urbanization trends over the past century were combined to examine the relationships seen over a range of spatial and temporal scales.

**Results/Conclusions:**

A consistent pattern of increased urbanization coincident with decreasing malaria transmission and elimination over the past century was found. Whilst it remains challenging to untangle whether this increased urbanization resulted in decreased transmission, or that malaria reductions promoted development, the results point to a close relationship between the two, irrespective of national wealth. The continuing rapid urbanization in malaria-endemic regions suggests that such malaria declines are likely to continue, particularly catalyzed by increasing levels of direct malaria control.

## Background

The range of malaria has contracted through a century of economic development and disease control [1-3]. During an era of renewed interest in elimination and eradication there is a need to better understand and quantify the forces behind this recession. A variety of direct control efforts were likely a major factor behind the global contraction in malaria transmission [2,3], although these gains were often coincident with rapid economic and social development and land use changes [4-6]. One major aspect of this development that has been shown to significantly impact malaria transmission is urbanization [7-9].

The process of urbanization includes physical landscape modification and transformation of environs through demand for resources and improved communications. Moreover, urbanization involves significant socio-economic change; generally improved health, housing and increased wealth [10,11]. These factors, common to urban areas, cause marked entomological, parasitological and behavioural effects that result in reduced malaria transmission both within the urban core and surrounding peri-urban areas [7,9,12]. A century of rapid urbanization has therefore likely had an impact on malaria transmission globally, but the size and importance of this impact has never been examined.

The recent construction of 20th Century time series of global urban extent data [13], and a contemporary malaria transmission map [14] that is comparable to historic data [15], enables a more detailed exploration of this relationship. Here, these datasets were utilized to examine for the first time the association at global, national and subnational scales between changes in malaria transmission and urban growth across the last century.

## Methods

### Data

#### Urbanization

**Figure 1:**
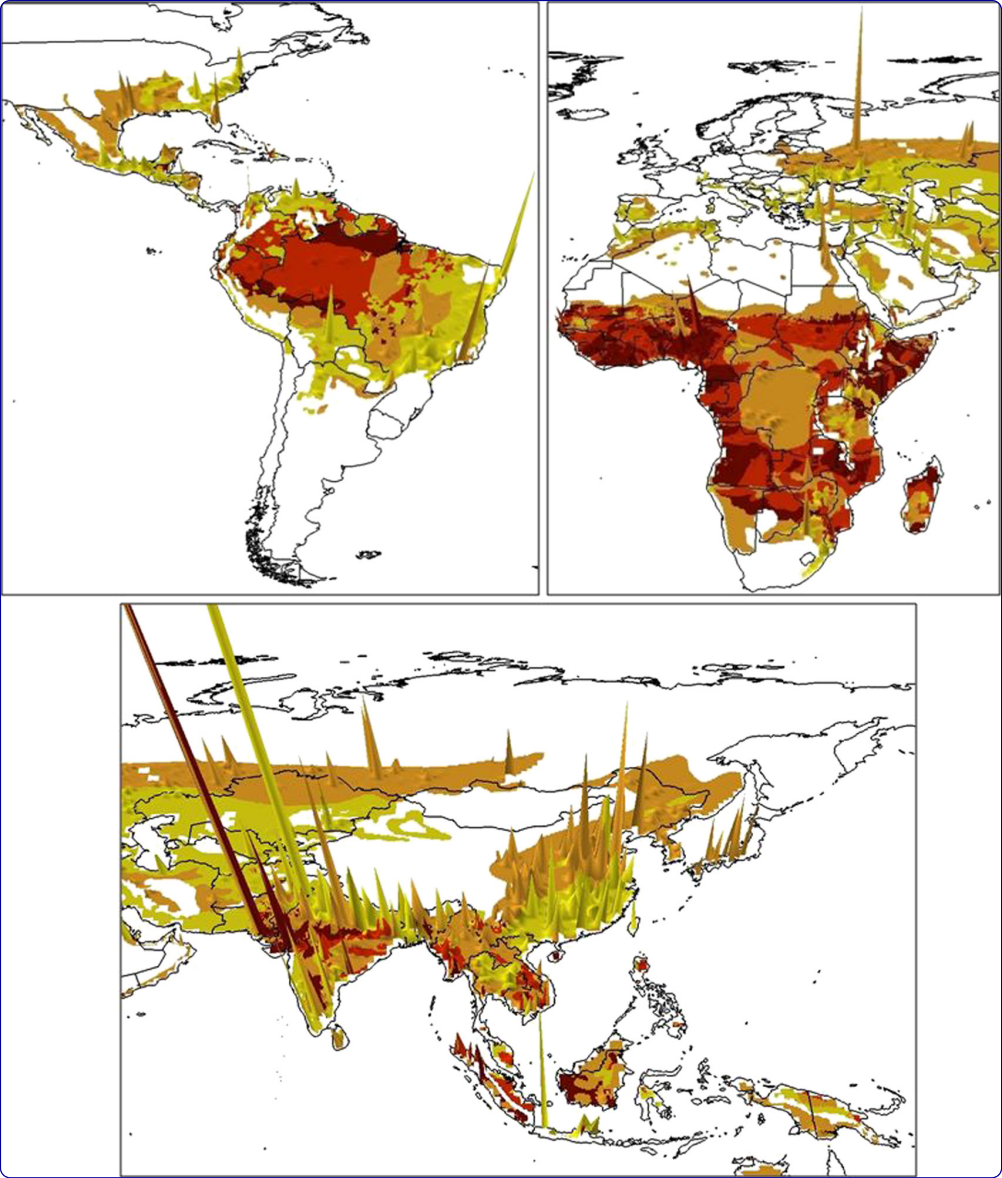
**Maps showing global changes in urban population size 1900-2000 and change in malaria endemicity.** The bar height is proportional to the size of urban population change. Areas that saw no change or an increase in endemicity are coloured dark red, those that saw a reduction by one endemicity class are in red, two classes in orange and three or more classes in yellow.

HYDE 3.1 [13,16] is a dynamic modelling effort of long-term historical population growth, providing a consistent database of 20th-century population and urban distribution at 5-minute spatial resolution (equivalent to approximately 10 km at the Equator). Full details of dataset construction are provided in Goldwijk *et al.*[13,16]. In brief, urban-rural population numbers and fractions for each country and for each decade were obtained from a variety of sources, including the UN [17] and published databases and reviews [18]. Urban areas were then mapped by combining different spatial datasets [19-21] and contemporary urban population datasets, which were derived from existing databases [21,22]. Finally, urban population densities over time were estimated based on the fitting of asymmetric Gaussian probability density function models to the population data [13]. The range of input datasets, assumptions and modeling methods used in construction of the HYDE 3.1 datasets mean that significant uncertainties are inherent in the outputs, and these are likely higher in the mapping covering both the early 20th Century and lower income areas of the World, where data scarcity meant that more assumptions had to be made [13,16]. Nevertheless, at the broad spatial and temporal scales of analysis undertaken here, the effects of such assumptions and uncertainties on results are likely small. Figure 1 shows changes in urban population sizes across the malaria endemic world from 1900 to 2000. Additional file 1 shows the mapped urban areas for 1900 and 2000, while Additional file 2 maps the change in urban extent over the 100-year period, and Additional file 3 shows urban extents in 1900 and 2000 for Brazil.

#### Malaria endemicity

The only global map of pre-intervention malaria endemicity dates from a 1968 study [15,23] in which a major synthesis of historical records, documents and maps of a variety of malariometric indices for all four *Plasmodium* species was used to map parasite rate (PR—the proportion of individuals with malaria parasites detectable in their peripheral blood) and stratified into four endemic classes associated with *Plasmodium falciparum* endemicity [24] (hypo-endemic, PR < 10%; meso-endemic, PR > 10% and <50%; hyperendemic, PR > 50% and < 75%; holo-endemic, PR > 75%). This map is the only reconstruction of historical malaria at its assumed historical peak around the start of the 20th Century and triangulates well with the plethora of national level malaria maps published throughout the last century [25]. The historical malaria endemicity map was scanned from the original publication, digitized on-screen and rasterized to a 5 × 5 km grid. The map for an area of Brazil is shown in Additional file 3(a) – the full map can be seen in Gething *et al.*[3].

The publication of an evidence-based map of contemporary malaria endemicity [14] and its conversion to classes that match the c.1900 map [3] described above, allows an audit of changes in the global epidemiology of malaria since the start of the 20th Century. With just two timepoints of malaria endemicity data, precise information on the timing and progression of changes is absent, placing a limitation on the conclusions that can be drawn from analyses. However, the datasets do provide a unique and valuable quantitative picture of the spatial changes in malaria epidemiology that have occurred over the last 100 years. The map of contemporary malaria endemicity was generated from a recently defined model of age-standardized *P. falciparum* parasite rate, *Pf*PR2-10 [26]. Using a model-based geostatistical framework, the underlying value of *Pf*PR2-10 at each location was modelled for the year 2007 as a transformation of a space-time Gaussian process (GP), with the number of *P. falciparum*-positive individuals in each survey modelled as a binomial variate given the unobserved age-standardized prevalence surface [14]. The GP was parameterized by a mean component and a space-time covariance function which was spatially anisotropic, used great-circle distance to incorporate the curvature of Earth, and included a periodic temporal component to capture seasonality. Bayesian inference was implemented using Markov chain Monte Carlo and direct simulation to generate posterior predictive samples of the 2007 annual mean prevalence surface and to assign each pixel to the endemicity class with the highest posterior probability of membership. This dataset for an area of Brazil is shown in Additional file 3(b). The classes matched those of the c.1900 endemicity map, enabling a class change dataset to be produced, which is shown in Figure 1 and Additional file 4.

In order to compare the observed changes in endemicity between these two time periods with levels of urbanization, it was necessary to translate both the historical and contemporary maps into approximate units of *Pf*R_C_, the *P. falciparum* basic reproductive number under the levels of control that existed at the time. This enabled comparisons of the effect sizes of changes in endemicity, following previous studies [3], and was undertaken using a simple *P. falciparum* transmission model [27] to estimate a value of *Pf*R_C_ corresponding to the mid-value of each endemicity class. Using these conversion values, maps of *Pf*R_C_ were made corresponding to both historical and contemporary endemicity. These two maps were overlaid in a geographical information system (GIS) (ArcGIS 9.2, ESRI Inc, Redlands CA, USA), and the relative change in *Pf*R_C_ between the historic and contemporary map was calculated for each 5 × 5 km pixel. These relative changes were summarized as areas of increase, areas of no change, and areas of decrease of between zero and one, one and two and greater than two orders of magnitude (see Additional file 5).

Finally, the recent construction of an evidence-based map of the limits of *Plasmodium vivax* transmission [28] meant that analyses could be repeated to examine the similarity in results between the two parasite species. Additional file 6 shows the *P. vivax* transmission limits map, constructed using data on the presence of *P. vivax* infection and spatial information on climatic conditions that impede transmission (low ambient temperature and extremely arid environments) in order to delineate areas where transmission was unlikely to take place.

#### Analyses

The data analyses focused on statistical explorations of the link between urbanization and changes in malaria endemicity. They covered three areas, examining in each case different spatial scales and factors:

(i) National scale analyses of differences in urbanization between countries that eliminated malaria during the past century and those that remain endemic today. Here, urban area percentages between 1900 and 2000 for those countries that remain endemic today, and urban area percentages between 1900 and the date of elimination (Additional file 7) for those countries that achieved it were compared.

(ii) Within country analyses of subnational differences in urbanization trends between regions that underwent malaria elimination 1900-2007, and regions within the same country that remain endemic today. Here, countries for which >20% of their land area became malaria-free over the past century and for which >20% of their land area remains endemic today were identified, and differences in urban areas, populations and rates of change between them were examined.

(iii)  Within country analyses of changes in transmission intensity over the past century and their relationship to urbanization trends. Here, per-country mean changes in transmission class in areas that are urban today, *versus* those that have remained rural, for malaria-endemic areas in 1900 were examined.

For (ii) and (iii), by undertaking analyses at the subnational scale and treating each country independently, the differences in wealth, governance and latitude between countries that are often confounding factors in assessing the drivers of long-term global changes in malaria endemicity are controlled for. For each set of analyses and each time period, the total urban areas and urban population counts were extracted using ArcGIS 9.3 (ArcGIS 9.2, ESRI Inc, Redlands CA, USA) and analysed using R2.10 [29].

## Results

**Figure 2:**
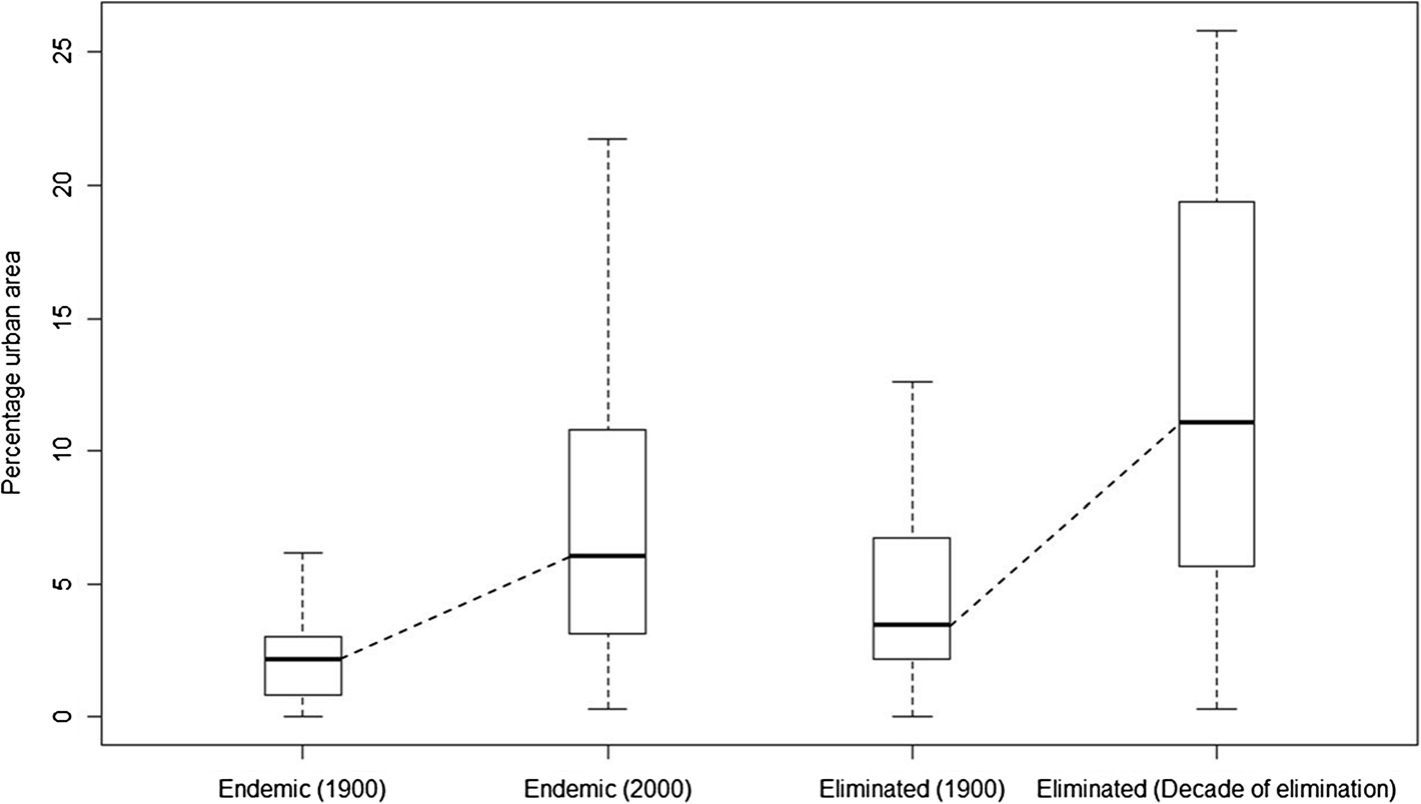
Box plots of the percentage urban area in 1900 and in 2000 for countries that are still endemic and in the decade that elimination was achieved for those countries that achieved elimination.

A consistent pattern of greater rates of urbanization in areas that either eliminated malaria or displayed the greatest transmission reductions was found. At the time of elimination certification of those countries that achieved malaria elimination, those areas that were originally malarious had significantly higher proportions (Mann-Whitney test: area, z = -3.207, p < 0.01; populations, z = -5.432, p < 0.01) of their populations living in urban areas and higher percentages of urban land area (Figure 2) than the contemporary situation in those areas of the world that remain endemic today. Moreover, the rate of urbanization from 1900 onwards was significantly larger in those countries that achieved elimination than those that remain endemic today (see Additional file 8). Those countries that achieved elimination exhibited significantly greater increases in the proportions of their populations (Mann-Whitney test: z = -1.985, p < 0.05) and land area (Mann-Whitney test: z = -3.232, p < 0.01) that became urban during the 20 years prior to elimination, compared to the recent rates of urbanization in currently endemic countries over the last 20 years. Such findings confirm the presence of a correlation between urbanization and the achievement of malaria elimination at national scales, but this relationship is likely to be intimately tied to wealth. Countries with greater wealth have greater malaria control resources at their disposal, and it is those wealthier countries that have succeeded in eliminating malaria, as confirmed by undertaking similar analyses based on average income (average income data from [30], Mann-Whitney test: z = -6.491, p < 0.01). Moreover, those higher income countries that succeeded in elimination are located in latitudinal regions of lower climatic and environmental receptivity to malaria transmission, making elimination likely an easier task with a fixed set of intervention tools. Therefore, untangling the effects of urbanization upon changes in malaria transmission required analysis independent of these economic or latitudinal biases, and these are described below.

**Figure 3:**
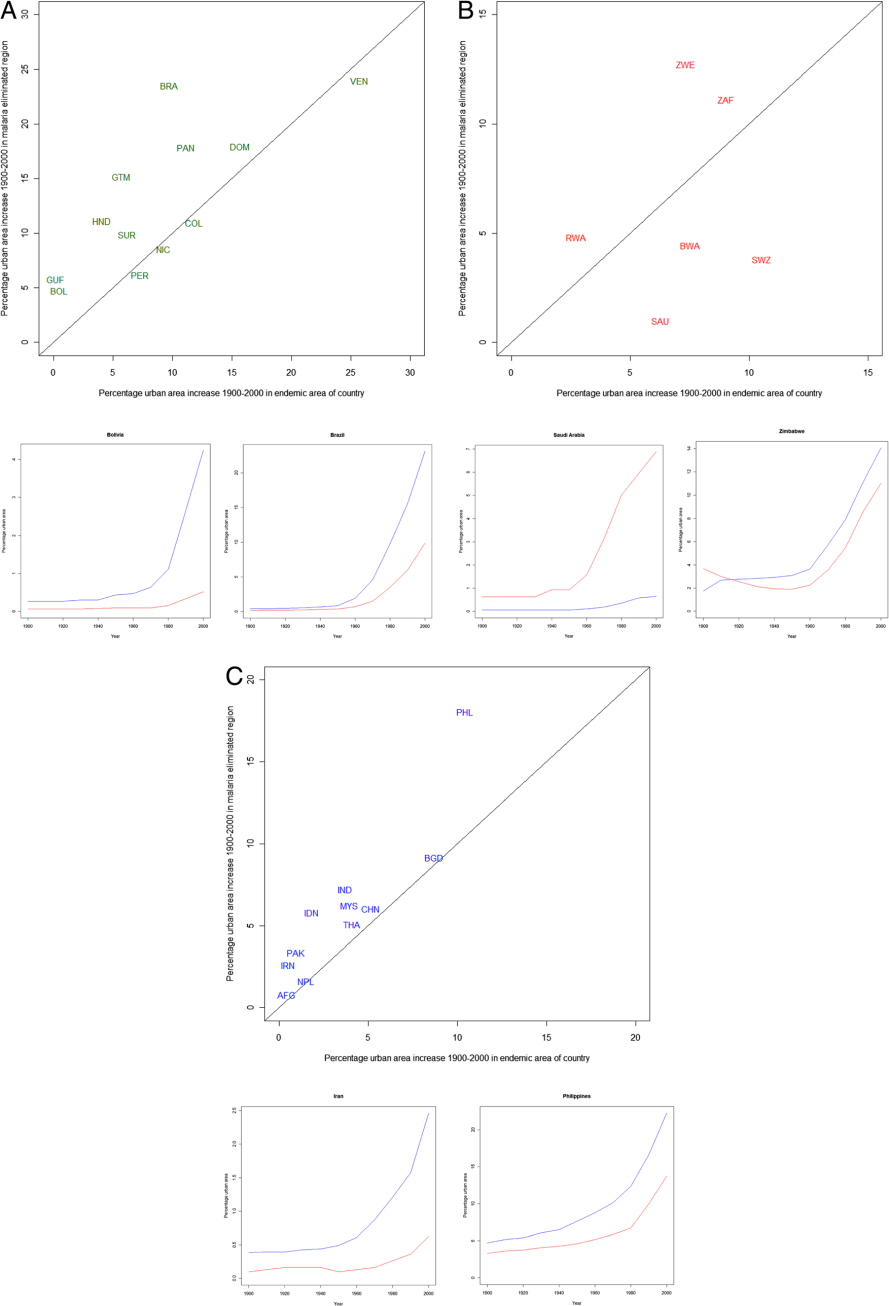
**Plots showing urban area changes 1900-2000 between areas of countries that remain malaria endemic today, and those that have undergone elimination for (a) the Americas, (b) Africa plus Arabian peninsula and (c) Asia.** In each case, scatterplots of urban area increases in endemic *versus* eliminated areas with one-to-one lines overlaid are shown at the top, and example plots of trends in urban area percentages between areas that eliminated malaria (blue) and that remain endemic (red) are shown at the bottom (the full set of these plots is provided in Additional file 9). The ISO country abbreviation for country name is used on the scatterplots ( http://www.iso.org/iso/english_country_names_and_code_elements).

Twenty-nine countries were identified for which at least a fifth of their land area became malaria-free over the past century and for which at least a fifth of their land area remains endemic today. Over 75% of these countries had greater proportions of land area urbanized in the malaria-free areas, and showed a greater percentage increase in urban extent (Figure 3) than the areas that remain endemic today. Both of these differences are significant (Wilcoxen test: urban area: z = -2.505 p < 0.05, urban extent change: z = -2.001, p < 0.05). However, these relationships show regional variations, (Figure 3). Almost all countries analysed in the Americas (Figure 3a) and Asia (Figure 3c) showed greater urbanization in their malaria-free *versus* still-endemic areas. In the Africa and Arabian Peninsula region (Figure 3b), where both levels of urbanization and transmission reductions were smaller, the results were more mixed. Many of those falling below the one-to-one line (Figure 3a- 3c) are countries where extremely arid or mountainous conditions have influenced human settlement to occur principally in areas most suitable for malaria transmission (e.g., Saudi Arabia, Botswana, Swaziland). A full set of national level results can be found in the Additional file 9. In addition, the results of a repeat analysis for *P. vivax* yielding very similar results are shown in Additional file 10.

Urban areas have been shown to exhibit lower levels of transmission than surrounding rural ones [8,9]. If the process of urbanization is a causal factor in malaria declines, a consistent pattern of greater transmission reduction in those areas that have undergone urbanization should be seen. Comparing mean changes in transmission class in areas that are urban today, *versus* those that have remained rural, for malaria-endemic areas in 1900, was possible in 158 countries. It shows that 82.3% underwent a greater transmission reduction in their urban areas than rural ones (Wilcoxen test: p < 0.001, see Additional file 10). Of the 28 countries that displayed a greater malaria reduction in rural areas, half of these were in sub-Saharan Africa, where the smallest levels of urbanization and reductions in transmission occurred. This confirms that greater transmission reductions occurred in areas that are urban today, but does not indicate if the largest declines were coincident with greater *rates* of urbanization.

**Figure 4:**
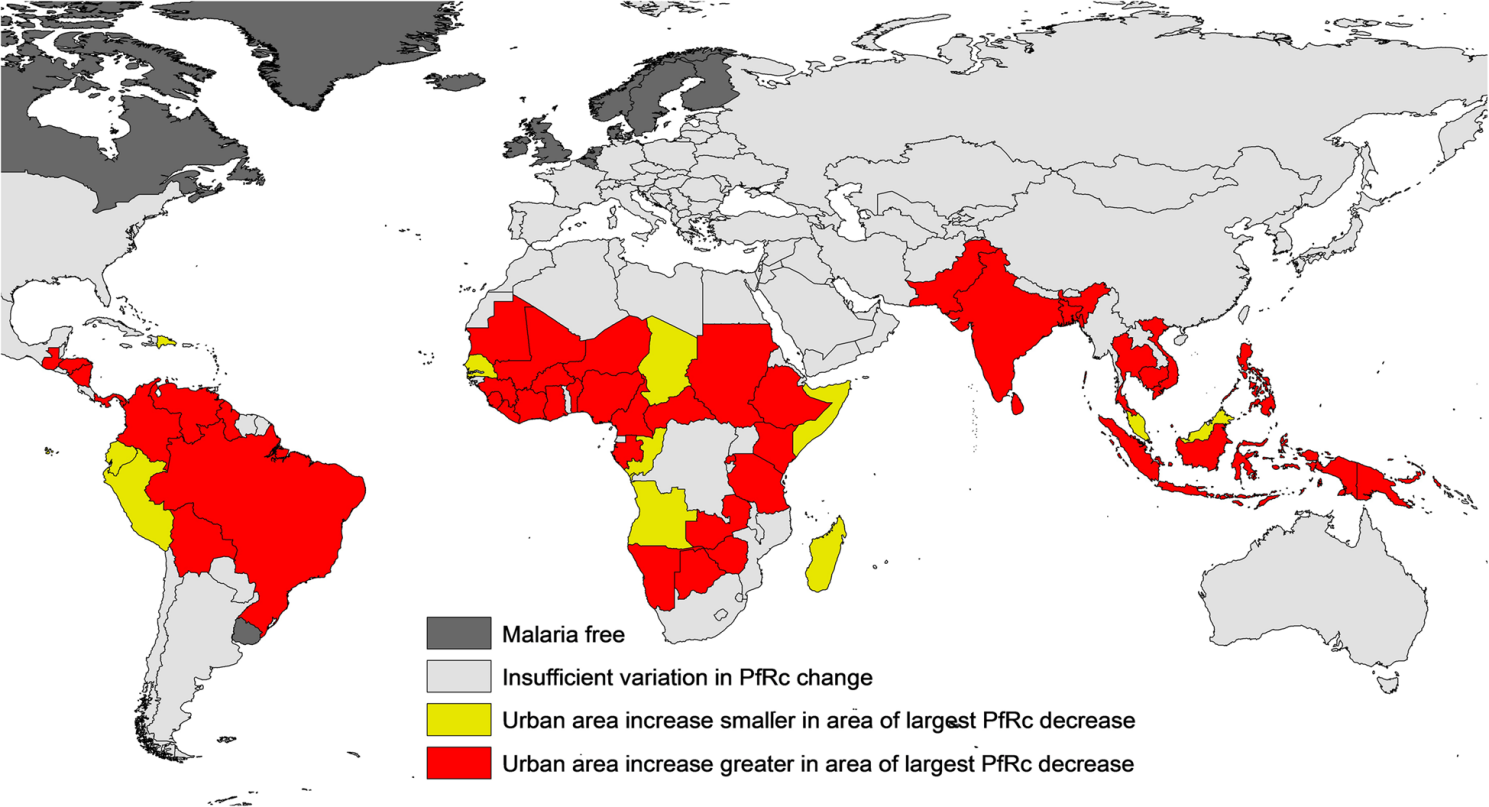
***Plasmodium falciparum *basic reproductive number (*PfR***_**C**_**) changes and urbanization.** Countries in red show a greater percentage increase in urban area over the past century in areas of the country with the greatest magnitude of *PfR*_C_ decreases than areas that showed the smallest changes. Countries in yellow show the reverse. Countries in light grey had either insufficient variation in *PfR*_C_ changes, or no transmission. Countries in dark grey have either always been malaria free, or only exhibited unstable transmission.

Finally, examination was undertaken of whether the rate of urbanization was higher in regions of countries that saw the greatest reductions in *Pf*R_C_. Sixty-three countries were identified that had two or more *Pf*R_C_ reduction classes, each covering at least 20% of their land area, and the rates of urbanization 1900-2000 in the areas exhibiting the smallest reduction in transmission were compared to those in the areas displaying the greatest reduction in transmission. Some 84.1% of countries displayed a greater urban increase in the areas that showed the greater *Pf*R_C_ reduction than those that showed the smallest transmission reductions (Wilcoxen test: p < 0.001, Figure 4). Further supporting analyses and more detail on these analyses are presented in Additional file 11.

## Discussion

The process of urbanization results in a variety of changes that reduce receptivity to malaria transmission [9-11]. Here, a clear picture of increased urbanization associated with greater malaria transmission reductions across countries and continents is documented for the first time. Whilst it remains challenging to untangle whether this urbanization resulted in decreased transmission, or that malaria reductions promoted development, a close relationship is evident, irrespective of national wealth and latitude.

Other local evidence [7,12,31,32] hints that changes commensurate with urbanization play a substantive role in driving malaria transmission declines. Whether measured by proportion of land area or population urbanized, the majority of nations that remain malaria endemic today exhibit substantially lower levels of urbanization compared to that at the time of elimination for those countries that have achieved it (Figure 2 and Additional file 8). Moreover, the magnitude of change in urban extent from 1900 is also correlated with malaria declines, with continental differences, notably sub-Saharan Africa showing lower levels of urbanization today, and smaller changes in urban extent and endemicity over the past century (Figure 3).

Recent malaria declines in sub-Saharan Africa point towards the success of interventions, however, in several cases the decline began before specific interventions were deployed, suggesting the contribution of alternative factors [33]. While malaria declines due to urbanization and its effects are likely to be more gradual than some of the sudden drops seen, it remains a possibility that the rapid urbanization ongoing in sub-Saharan Africa [34] is at least a contributory driver to these changes.

While over three-quarters of countries show decreasing transmission in areas that have undergone urbanization over the past century, a handful of countries go against this trend. A possible reason for this is the likely multi-factorial complexity behind both changes in transmission and human settlement dynamics, and the difficulty of attributing changes to a single cause. Recent analyses have indicated that vector-borne and parasitic diseases have systematically impacted economic growth [35], but more detailed studies of these types of relationships across disease types and ecozones are required to gain a fuller understanding. Almost all of the countries that show greater transmission reductions in rural areas are those where human settlement was constrained to the more malarious areas of the country, due to uninhabitable arid or mountainous areas elsewhere. Equally however, the reverse is true for some countries – i.e. uninhabitable areas with intense transmission forced human settlement in the lower transmission regions.

It is clear that various sources of uncertainty exist in the inputs and methodologies used in this study. Uncertainties are inherent in the urbanization datasets through the lack of data for many regions and time periods, and the assumptions made to fill these gaps [13]. The proposed levels of historical endemicity [15,23] are plausible when triangulated against other values reported from the pre-intervention era (for example, [26,36]), but the relatively crude categorization of all-cause malaria endemicity strata and the cartographic approach used preclude a more formal quantification of the global *P. falciparum* endemicity declines and their link to urbanization beyond the broad relationships presented here. Nevertheless, recent mapping of the limits of *P. vivax* transmission [28] and analyses of contemporary impacts of urbanization [8], indicate very similar effects and contractions as that seen for *P. falciparum*, with results repeated, where possible, for *P. vivax* showing almost identical results (Additional file 10), and transmission rarely more intense than meso-endemic [37,38].

Much of the low-income world, where malaria burden is highest and levels of urbanization are lowest, is set to undergo an urban and demographic transition in the coming decades [17,34], ultimately likely arriving at levels of urbanization similar to those exhibited by countries that eliminated malaria (Additional file 8). Significant efforts towards modelling future malaria scenarios have been completed or are underway, focused principally on the effects of a variety of interventions [39-41] or on climate change scenarios [42-45], but the impacts of urbanization are rarely considered [46]. Yet, if the past century of malaria declines are indicative, the study of its impacts should receive more attention as nations start to monitor their progress toward elimination [47]. There exist multiple data gaps and uncertainties in obtaining suitable data to build urbanization into scenario models, however. Firstly, simple consistent definitions of what constitutes an urban area in general are difficult to outline. Beyond this, multiple approaches to mapping urban extents in a consistent fashion have been attempted (e.g., [48-50]), but the spatial modelling of their future growth is lacking. Secondly, definitions and measures of urbanization that are relevant to understanding transmission patterns are poorly quantified, with only occasional attempts at empirical definitions made [8,9], and little consideration of the urban preferences of the dominant *Anopheles* species [51-53], or adaptation of them to urban environments [54,55]. Finally, the treatment of urban areas as single homogenous entities ignores the great variations in demographic, socio-economic and land uses within cities with, for example, urban agricultural practices often maintaining transmission within urban areas [56-58].

The quantification of a global recession in the range and intensity of malaria over the 20th century has allowed us to review the impact that urbanization has had on these declines, and gauge its importance as a driver of future changes in malaria epidemiology. Results highlight for the first time the consistent relationship between urbanization and malaria declines over the past century globally, and point towards continuing declines as urbanization permanently alters the receptivity of many areas to malaria transmission. The findings presented here suggest that these trends will likely continue to catalyze malaria declines on the path to the goal of a malaria-free future.

## Competing interests

The authors declare that they have no competing interests.

## Authors’ contributions

AJT conceived the analyses, developed the study design and conducted the analyses. PWG, DLS and SIH provided the malaria endemicity data and mathematical models for dataset conversions. All authors contributed to the writing of the manuscript. All authors read and approved the final manuscript.

## Supplementary Material

Additional file 1 **Urban extents and malaria transmission mapped for 1900 and 2000.** Description: Maps of urban extents in 1900 and 2000 overlaid onto mapped areas of where malaria was eliminated over the past century and where it remains endemic today. Click here for file

Additional file 2 **Change in urban extent between 1900 and 2000.** Description: Map of estimated changes in urban extent globally between 1900 and 2000. Click here for file

Additional file 3 **Malaria endemicity classes and urban areas in Brazil.** Description: Maps of malaria endemicity classes and urban areas in Brazil for 1900 and 2000. Click here for file

Additional file 4 **Change in malaria endemicity class between 1900 and 2007.** Description: Map of changes in malaria endemicity class between 1900 and 2007. Click here for file

Additional file 5 **The magnitude of decrease in *P. falciparum *basic reproductive number 1900-2007.** Description: Map of the magnitude of decrease in *P. falciparum* basic reproductive number for 1900-2007. Click here for file

Additional file 6 **The global spatial limits of *Plasmodium vivax *malaria transmission in 2009.** Description: Map of the global spatial limits of *Plasmodium vivax* malaria transmission in 2009. Click here for file

Additional file 7 **Dates of malaria elimination for those countries that achieved it.** Description: Table showing the estimated and official dates of malaria elimination for those countries that achieved it. Click here for file

Additional file 8 **Urbanization and countries that have eliminated malaria.** Description: Description and results of statistical analyses of urbanization levels between countries that achieved elimination and those that remain endemic today. Click here for file

Additional file 9 **Urbanization and sub-national malaria elimination.** Description: Description and results of per-country statistical analyses of urbanization levels between regions that became malaria free and those that remain endemic today. Click here for file

Additional file 10 **Results using the contemporary limits of *Plasmodium vivax *transmission.** Description: Description and results of analyses repeated using *Plasmodium vivax* transmission limits data. Click here for file

Additional file 11 **Urbanization and changes in malaria transmission.** Description: Description and results of per-country statistical analyses of changes in malaria transmission intensity and urbanization levels. Click here for file
