# Supplementary material for: Urbanization and the global malaria recession
Source: Malar J. 2013 Apr 17;12:133. doi: 10.1186/1475-2875-12-133 (PMC3639825; doi:10.1186/1475-2875-12-133)
Supplement: Additional file 1 — Urban extents and malaria transmission mapped for 1900 and 2000. Description: Maps of urban extents in 1900 and 2000 overlaid onto mapped areas of where malaria was eliminated over the past century and where it remains endemic today. [file 1475-2875-12-133-S1.pdf]

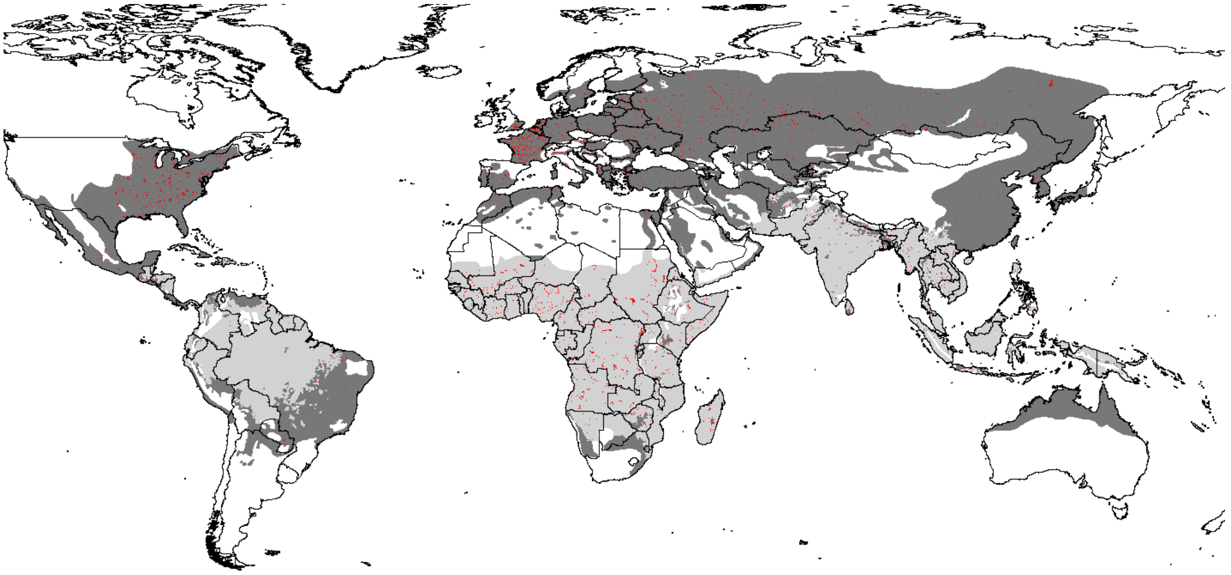

(a)

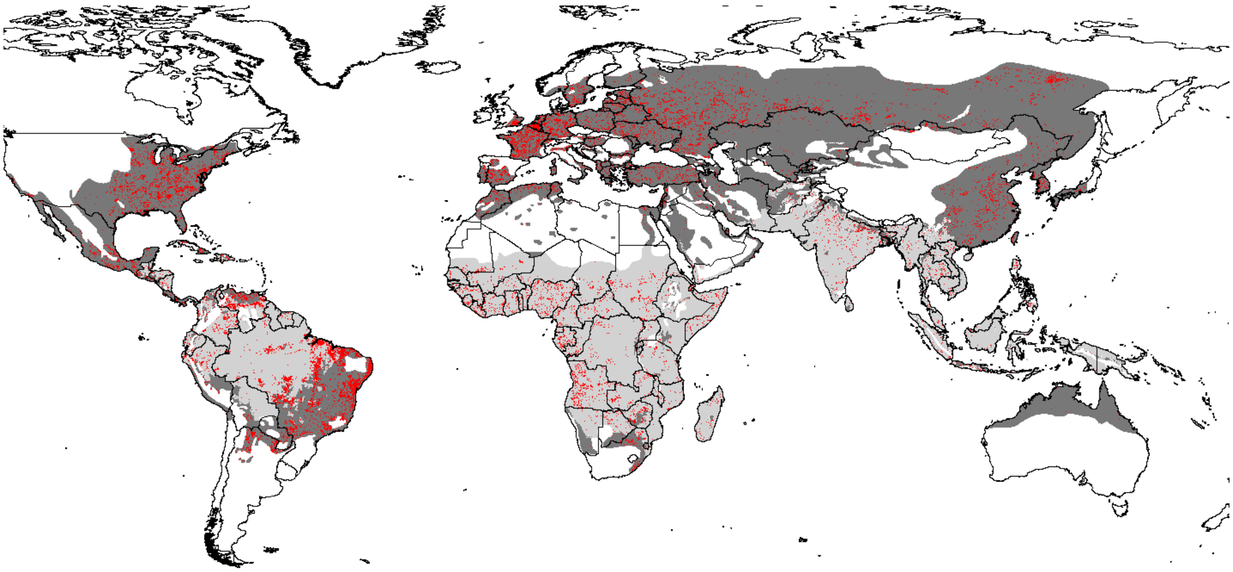

(b)

Urban extents mapped in red for (a) 1900, and (b) 2000. These are shown on top of areas where malaria was eliminated between 1900 and 2007 (dark grey) and areas where transmission continues today (light grey).
