# Supplementary material for: Urbanization and the global malaria recession
Source: Malar J. 2013 Apr 17;12:133. doi: 10.1186/1475-2875-12-133 (PMC3639825; doi:10.1186/1475-2875-12-133)
Supplement: Additional file 2 — Change in urban extent between 1900 and 2000. Description: Map of estimated changes in urban extent globally between 1900 and 2000. [file 1475-2875-12-133-S2.pdf]

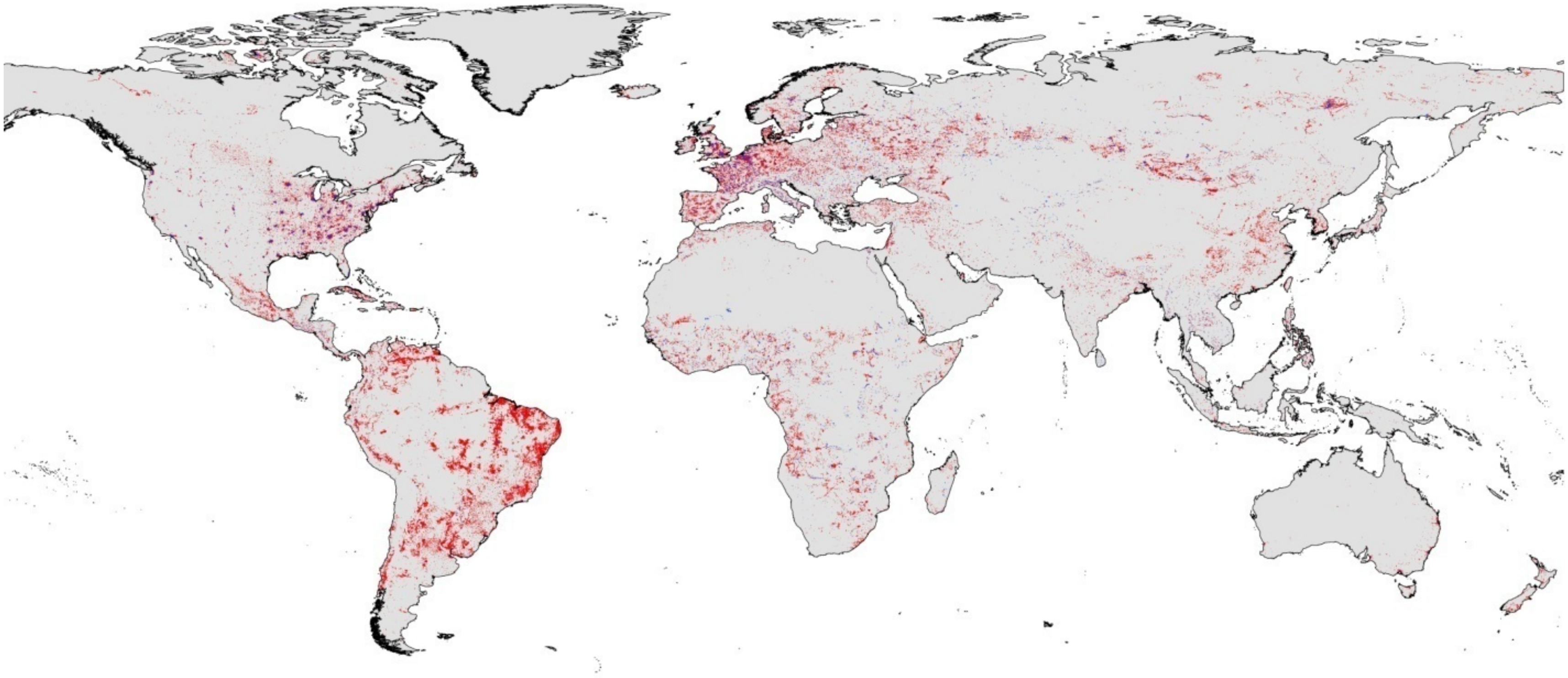

Change in urban extent between 1900 and 2000. Blue denotes urban areas existing in 1900, red denotes those that have developed since.
