# Supplementary figures and images for: Urbanization and the global malaria recession
Source: Malar J. 2013 Apr 17;12:133. doi: 10.1186/1475-2875-12-133 (PMC3639825; doi:10.1186/1475-2875-12-133)

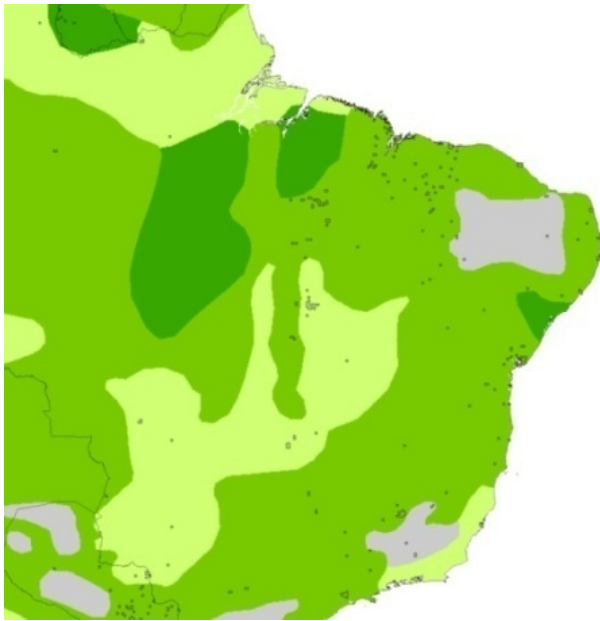

(a)

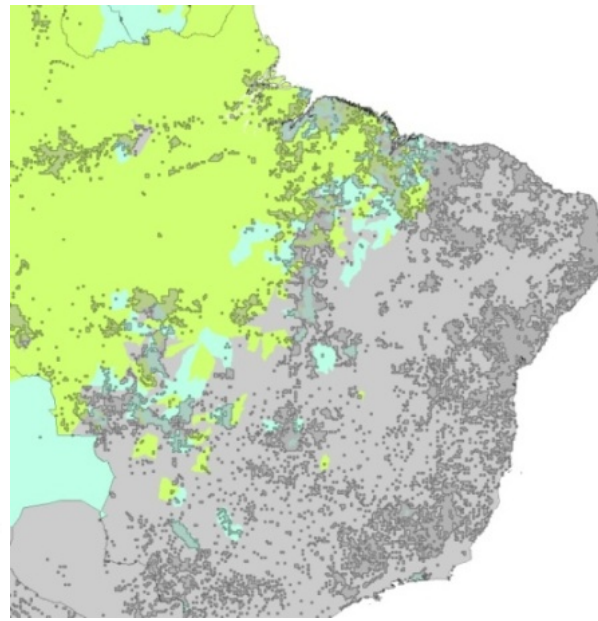

(b)

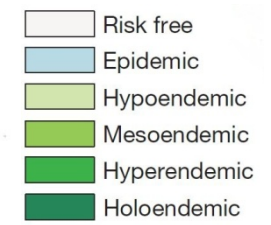

*Malaria endemicity classes and urban areas (outlined in grey) in Brazil for (a) 1900 and (b) 2000*

Supplement: Additional file 3 — Malaria endemicity classes and urban areas in Brazil. Description: Maps of malaria endemicity classes and urban areas in Brazil for 1900 and 2000. [file 1475-2875-12-133-S3.pdf]
