# Supplementary material for: Urbanization and the global malaria recession
Source: Malar J. 2013 Apr 17;12:133. doi: 10.1186/1475-2875-12-133 (PMC3639825; doi:10.1186/1475-2875-12-133)
Supplement: Additional file 4 — Change in malaria endemicity class between 1900 and 2007. Description: Map of changes in malaria endemicity class between 1900 and 2007. [file 1475-2875-12-133-S4.pdf]

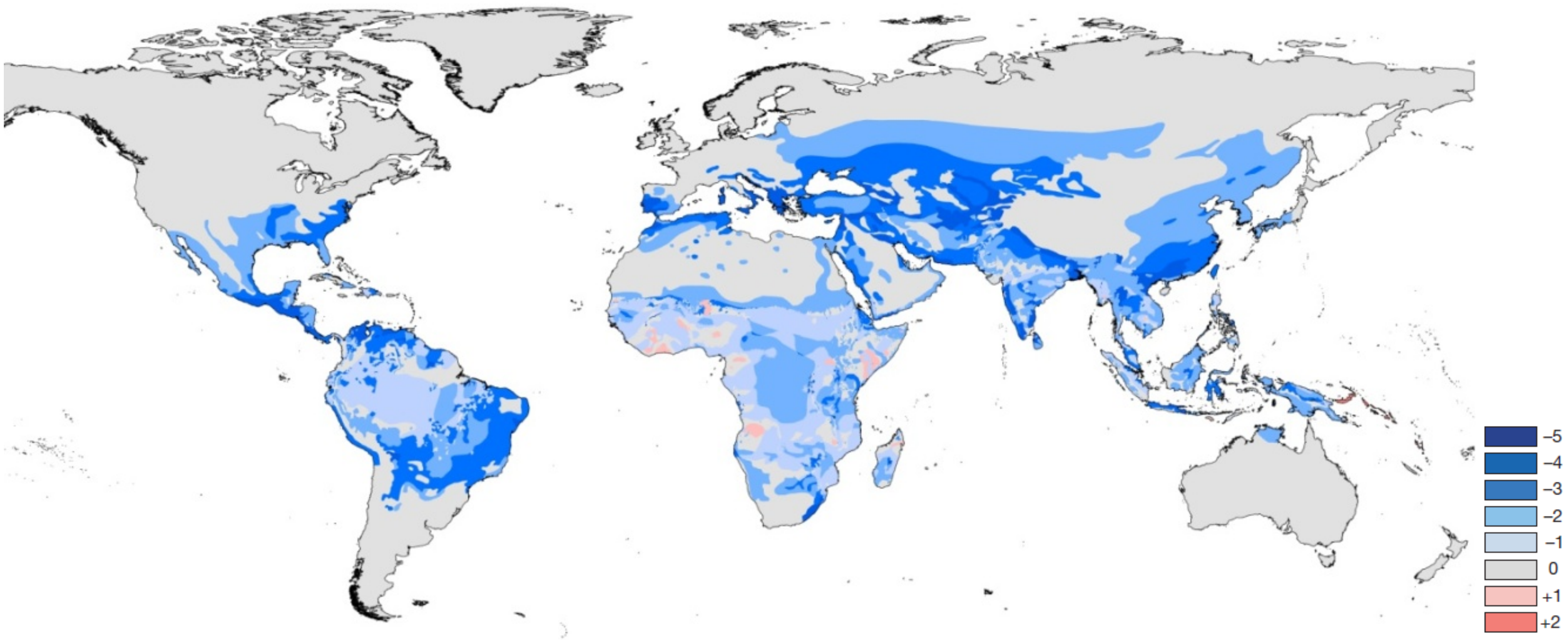

Change in malaria endemicity class between 1900 and 2007. Negative values denote a reduction in endemicity, positive values an increase.
