# Supplementary material for: Urbanization and the global malaria recession
Source: Malar J. 2013 Apr 17;12:133. doi: 10.1186/1475-2875-12-133 (PMC3639825; doi:10.1186/1475-2875-12-133)
Supplement: Additional file 5 — The magnitude of decrease in P. falciparum basic reproductive number 1900-2007. Description: Map of the magnitude of decrease in P. falciparum basic reproductive number for 1900-2007. [file 1475-2875-12-133-S5.pdf]

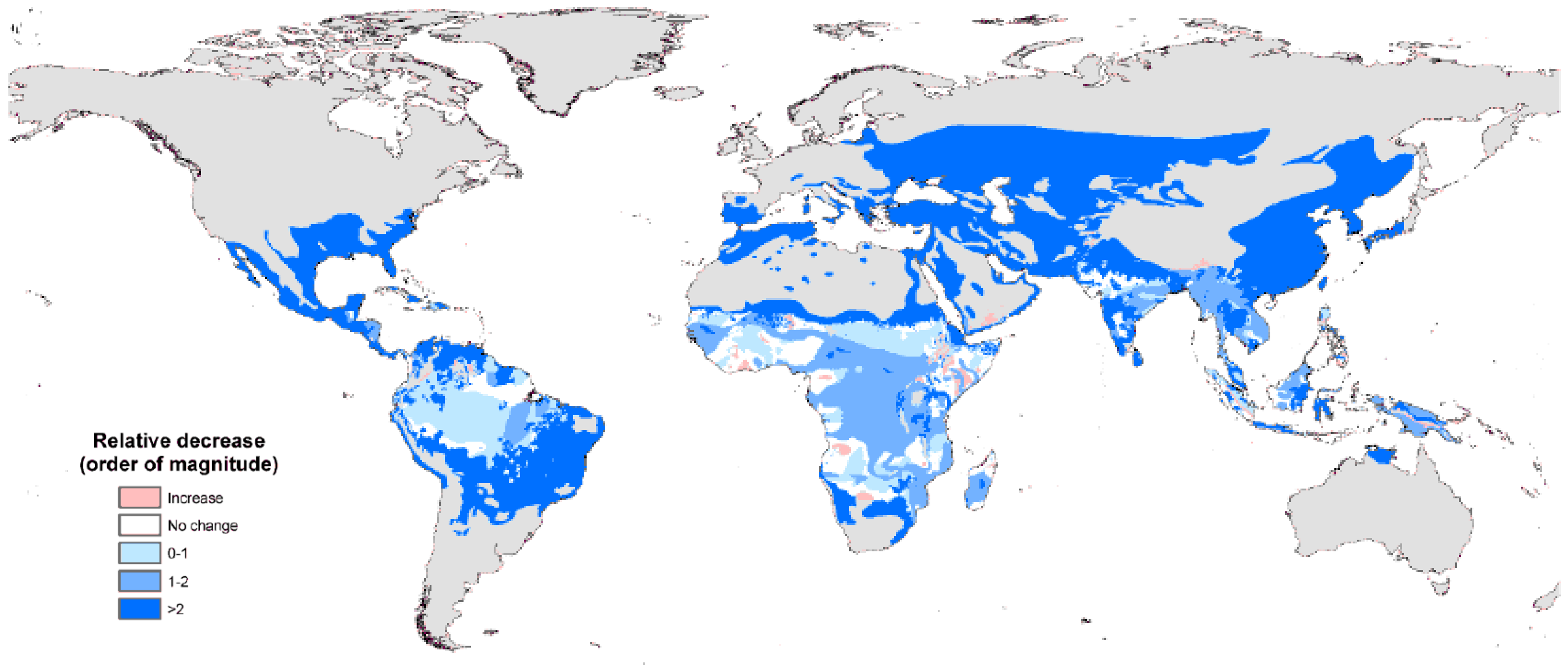

The magnitude of decrease in P. falciparum basic reproductive number (PfR0), adapted from Gething et al [1].

Reference

1. Gething PW, Smith DL, Patil AP, Tatem AJ, Snow RW, Hay SI: **Climate change and the global malaria recession.** *Nature* 2010, **465:**342-346.
