# Supplementary material for: Urbanization and the global malaria recession
Source: Malar J. 2013 Apr 17;12:133. doi: 10.1186/1475-2875-12-133 (PMC3639825; doi:10.1186/1475-2875-12-133)
Supplement: Additional file 6 — The global spatial limits of Plasmodium vivax malaria transmission in 2009. Description: Map of the global spatial limits of Plasmodium vivax malaria transmission in 2009. [file 1475-2875-12-133-S6.pdf]

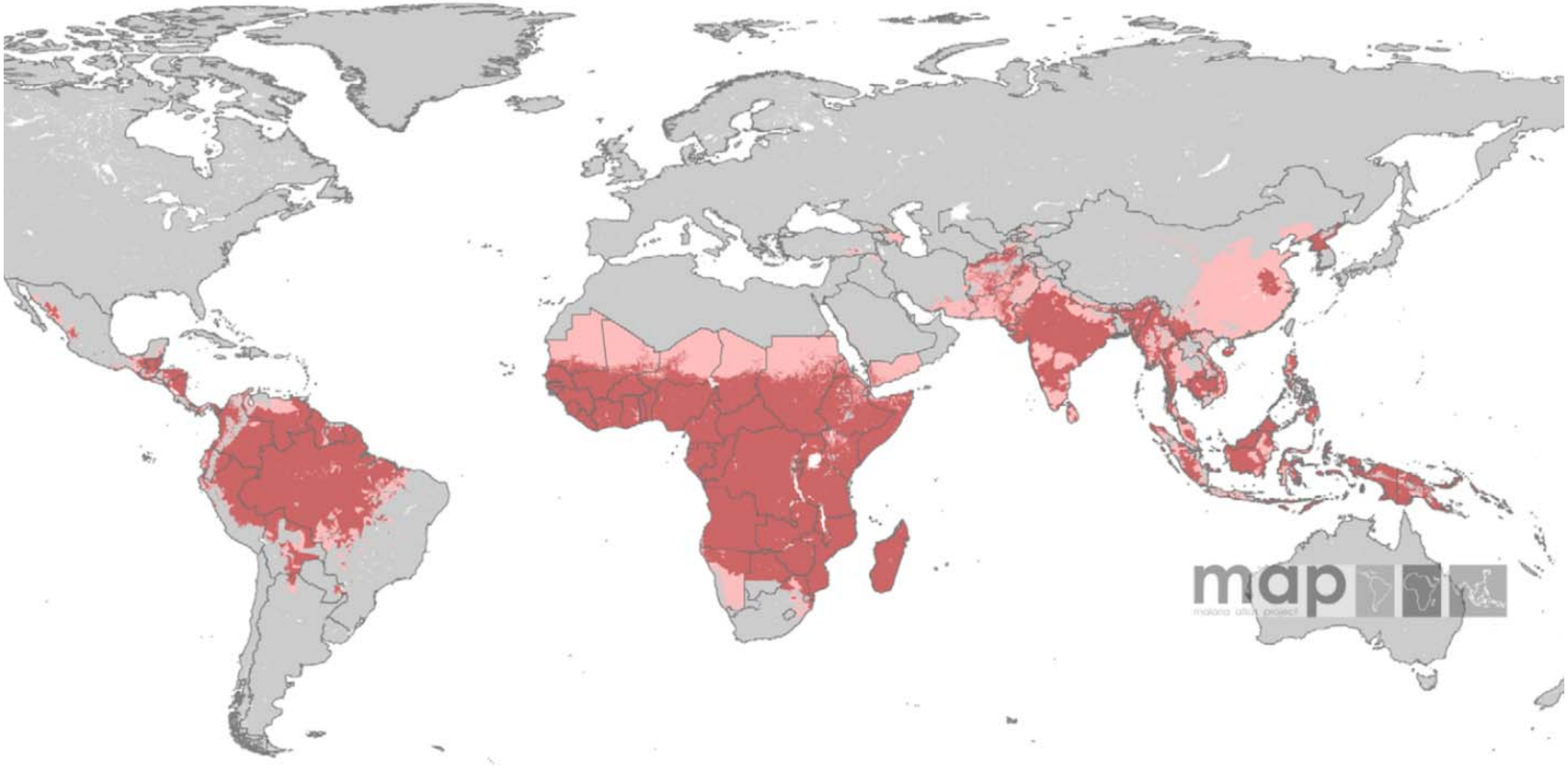

The global spatial limits of Plasmodium vivax malaria transmission in 2009 as defined in Guerra et al [1]. Red areas represent stable transmission, pink areas display unstable transmission and grey areas are P. vivax free.

References

1. Guerra CA, Howes RE, Patil AP, Gething PW, Van Boeckel TP, Temperley WH, Kabaria CW, Tatem AJ, Manh BH, Elyazar IRF, et al: **The international limits and population at risk of *Plasmodium vivax* transmission in 2009.** *PLoS Negl Trop Dis* 2010, **4:**e774.
