# Supplementary material for: Urbanization and the global malaria recession
Source: Malar J. 2013 Apr 17;12:133. doi: 10.1186/1475-2875-12-133 (PMC3639825; doi:10.1186/1475-2875-12-133)
Supplement: Additional file 7 — Dates of malaria elimination for those countries that achieved it. Description: Table showing the estimated and official dates of malaria elimination for those countries that achieved it. [file 1475-2875-12-133-S7.pdf]

Country	Elimination date
Andorra	1945-1970
Anguilla	malaria free, no dates of elimination given
Antigua	malaria free, no dates of elimination given
Aruba	malaria free, no dates of elimination given
Australia	1986
Austria	1951
Bahamas	malaria free, no dates of elimination given
Bahrain	1979
Barbados	malaria free, no dates of elimination given
Belarus	1970-1990
Belgium	1945-1970
Bermuda	malaria free, no dates of elimination given
Bosnia and Herzegovina	1945-1970
Brunei Darussalam	1987
Bulgaria	1965
Canada	1950
Cayman Islands	malaria free, no dates of elimination given
Chile	1975
China (Taiwan)	1965
Croatia	1956-1970
Cuba	1967
Cyprus	1953
Czech Republic	1953
Denmark	1950
Dominica	Between 1965-1967
Estonia	1952
Falkland Islands (Malvinas)	pre-1900
Faroe Islands	pre-1900
Finland	1952
France	1952
French Polynesia	pre-1900
Gaza Strip	1965
Germany	1951
Greece	1945-1970
Greenland	pre-1900
Grenada	Between 1961-1963
Guadeloupe	malaria free, no dates of elimination given
Hong Kong	1945-1970
Hungary	1963
Iceland	pre-1900
Ireland	
Italy	Between 1967_1970

Jamaica	1958
Japan	1950
Jordan	1970
Kazakhstan	2000
Korea, South	1990-2008
Kuwait	no cases since 1979
Latvia	1952
Lebanon	1963
Lesotho	Originally malaria free
Libya	1973
Lithuania	1952
Luxembourg	1945-1970
Maldives	malaria free, no dates of elimination given
Martinique	malaria free, no dates of elimination given
Mauritius	1963
Moldova, Republic of	1970-1990
Monaco	1945-1970
Mongolia	pre-1900
Montserrat	malaria free, no dates of elimination given
Morocco	2005
Netherlands	1967-1970
New Zealand	pre-1900
Norway	pre-1900
Oman	2004
Philippines	2006
Poland	1965-1967
Portugal	1973
Puerto Rico	1962
Qatar	1970
Reunion	1979
Romania	1967
Russia	1970-1990
Saint Lucia	1961-1963
San Marino	1945-1970
Serbia and Montenegro	1945-1970
Seychelles	malaria free, no dates of elimination given
Singapore	1986
Slovakia	1953
Slovenia	1945-1970
Spain	1964
St. Kitts & Nevis	malaria free, no dates of elimination given
St. Vincent & Grenadines	malaria free, no dates of elimination given
Svalbard and Jan Mayen	Originally malaria free

Islands	
Sweden	pre-1900
Switzerland	pre-1900
Syrian Arab Republic	2005
The former Yugoslav Republic	Post WWII
Trinidad and Tobago	1965 (malaria returned, but eliminated again later)
Tunisia	1979
Turks & Caicos Islands	malaria free, no dates of elimination given
U.K. of Great Britain and N	1952
Ukraine	1970-1990
United Arab Emirates	1990-2008
United States of America	1951
Uruguay	Pre-1900
West Bank	1965

Dates of malaria elimination for those countries that achieved it. Historical literature, review papers and personal communications were collated to create a combined record of the dates that malaria elimination was achieved for each country.
