# Supplementary material for: Urbanization and the global malaria recession
Source: Malar J. 2013 Apr 17;12:133. doi: 10.1186/1475-2875-12-133 (PMC3639825; doi:10.1186/1475-2875-12-133)
Supplement: Additional file 8 — Urbanization and countries that have eliminated malaria. Description: Description and results of statistical analyses of urbanization levels between countries that achieved elimination and those that remain endemic today. [file 1475-2875-12-133-S8.pdf]

Urbanization and countries that have eliminated malaria

Figure 2 in the main paper shows a boxplot comparing (i) urban area percentages between 1900 and 2000 for those countries that remain endemic today, and (ii) urban area percentages between 1900 and the date of elimination for those countries that achieved it (see Additional file 7 for dates). In 1900, both the proportion of land area (figure 2, main paper) and population that were urban were similar for the malarious areas of those countries that eventually eliminated malaria and those that remain endemic today. The differences seen were only weakly significant (Mann-Whitney test: area, $z = -2.543$, $p<0.05$; populations, $z=-2.503$, $p<0.05$). However, at the time of elimination certification of those countries that achieved malaria elimination, those areas that were originally malarious had significantly higher proportions (Mann-Whitney test: area, $z = -3.207$, $p<0.01$; populations, $z=-5.432$, $p<0.01$) of their populations living in urban areas and higher percentages of urban land area (figure 2, main paper) than the contemporary situation in those areas of the world that remain endemic today. The simple plot in figure 2 of the main paper shows that those countries that successfully eliminated had, at the time of elimination, a significantly higher proportion of their land area urbanized than those countries that are still malaria endemic do today. Moreover, the rate of urbanization from 1900 onwards was significantly larger in those countries that achieved elimination than those that remain endemic today.

Analyses were also undertaken focused on comparing urban area and urban population changes in the 20 years prior to elimination for those countries that achieved elimination against the 1980-2000 period for currently endemic countries to examine whether rates of urbanization immediately before elimination was achieved were significantly higher. Results showed that those countries that achieved elimination exhibited significantly greater increases in the proportions of their populations (Mann-Whitney test: $z = -1.985$, $p<0.05$) and land area (Mann-Whitney test: $z = -3.232$, $p<0.01$) that became urban during the 20 years prior to elimination, compared to the recent rates of urbanization in currently endemic countries over the last 20 years.

These results confirm the presence of a correlation between urbanization and the achievement of malaria elimination at national scales, but this relationship is likely to be intimately tied to wealth. Countries with greater wealth have greater malaria control resources at their disposal, and it is those wealthier countries that have succeeded in eliminating malaria, as confirmed by undertaking similar analyses based on average income (Average income data from http://www.gapminder.org/data/, Mann-Whitney test: $z = -6.491$, $p < 0.01$). Moreover, those higher income countries that succeeded in elimination are located in latitudinal regions of lower climatic and environmental receptivity to malaria transmission, making elimination an easier task with a fixed set of intervention tools. Therefore, untangling the effects of urbanization upon changes in malaria transmission requires analysis independent of these economic or latitudinal biases. The subsequent additional files present subnational analyses that circumvent these issues.
