# Supplementary material for: Urbanization and the global malaria recession
Source: Malar J. 2013 Apr 17;12:133. doi: 10.1186/1475-2875-12-133 (PMC3639825; doi:10.1186/1475-2875-12-133)
Supplement: Additional file 9 — Urbanization and sub-national malaria elimination. Description: Description and results of per-country statistical analyses of urbanization levels between regions that became malaria free and those that remain endemic today. [file 1475-2875-12-133-S9.pdf]

Urbanization and sub-national malaria elimination

In addition to those countries that have eliminated malaria completely from their borders, many countries that remain endemic today saw substantial parts of their territories become malaria free over the last century (e.g. Brazil in Additional file 3). These countries provide opportunities to examine the subnational relationships between local malaria elimination and urbanization that are independent of wealth and latitudinal differences between countries.

Twenty-nine countries were identified for which >20% of their land area became malaria-free over the past-century and for which >20% of their land area remains endemic today. The choice of 20% is an arbitrary one designed to ensure that conclusions were not drawn for countries where a tiny proportion of their land was estimated to have changed or remained the same. Testing with thresholds of 10% and 30% resulted in little changes in overall results, showing that outputs were not highly sensitive to this choice. Figure 2 in the main document shows that 80% of the countries had greater proportions of land area urbanized in the malaria free areas, and showed a greater percentage increase in urban extent than the areas that remain endemic today. Both of these differences are significant (Wilcoxen test: urban area: $z=-2.505$ $p<0.05$, urban extent change: $z=-2.001$, $p<0.05$). Many of those falling below the one-to-one line are countries where extremely arid or mountainous conditions across much of the land area have driven human settlement to occur in the most suitable areas for malaria transmission, e.g. Namibia, Saudi Arabia, Botswana, Swaziland. Moreover, almost all those countries in the Americas and Asia showed greater urbanization in their malaria free areas, whereas for the Africa region, where both levels of urbanization and transmission reductions were smaller, it was a much more mixed picture.

Figure 9.1 below shows that the general trend for the majority of countries that have eliminated malaria from at least 20% of their territory, but remain endemic in at least 20% of their territory today, is of increased urbanization in those areas where malaria was eliminated from, compared to those areas that have remained endemic. There exist exceptions, though again, these are nearly all very sparsely populated arid countries (Afghanistan, Botswana, Namibia, Saudi Arabia), where population distribution is overdispersed into the only small areas that are habitable, and thus, support transmission. Once these are accounted for, the vast majority of remaining countries show increased urbanization in eliminated areas.

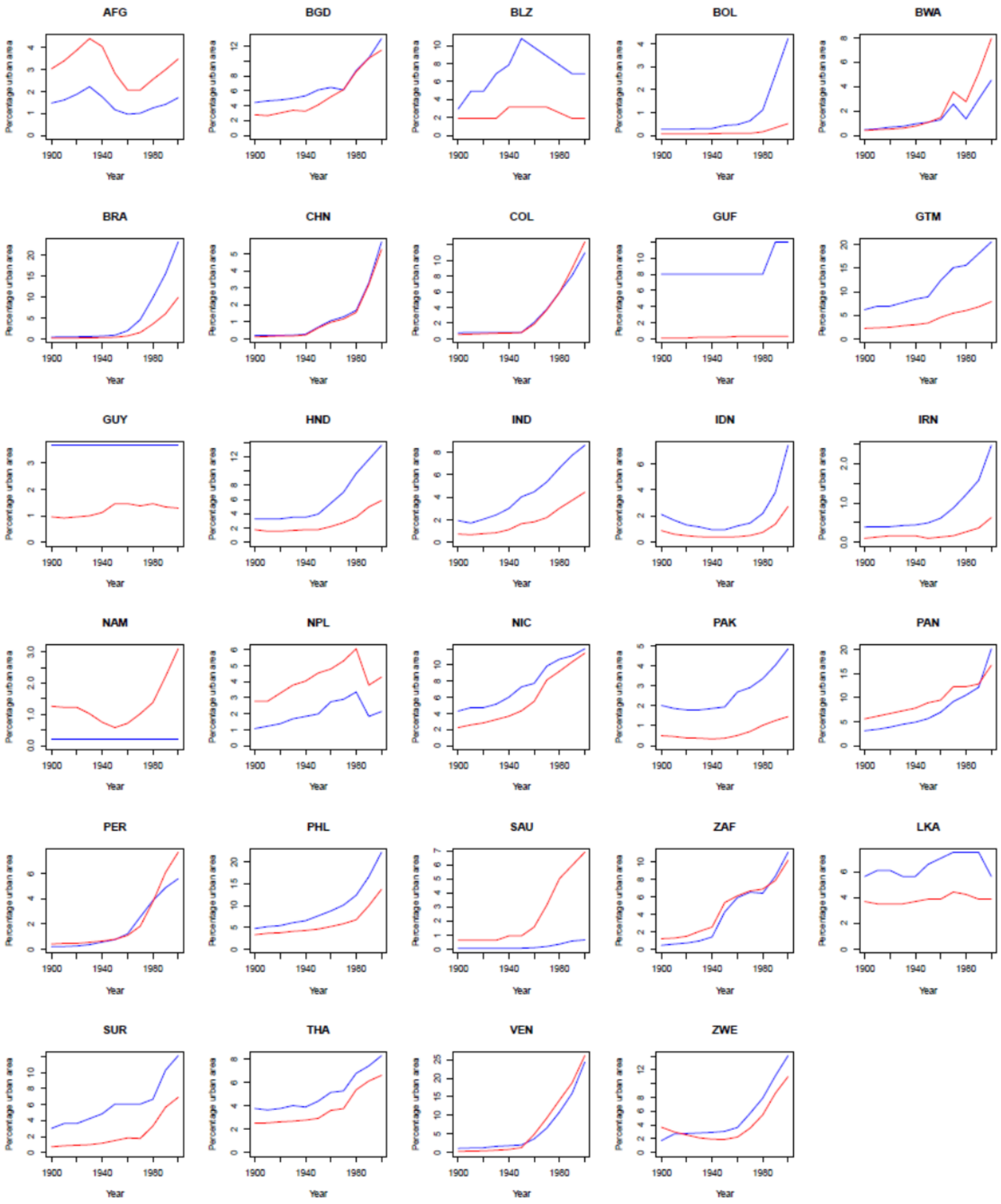

Figure 9.1. Plots of percentage of area that is classed as urban over time in (i) areas that underwent malaria elimination 1900-2007 (blue line) and (ii) areas that have remained endemic 1900-2007 (red line), for countries where substantial areas have become malaria free between 1900 and 2007. See http://www.worldatlas.com/aatlas/ctycodes.htm for country codes.
