# Supplementary material for: Urbanization and the global malaria recession
Source: Malar J. 2013 Apr 17;12:133. doi: 10.1186/1475-2875-12-133 (PMC3639825; doi:10.1186/1475-2875-12-133)
Supplement: Additional file 10 — Results using the contemporary limits of Plasmodium vivax transmission. Description: Description and results of analyses repeated using Plasmodium vivax transmission limits data. [file 1475-2875-12-133-S10.pdf]

Results using the contemporary limits of *Plasmodium vivax* transmission

The proposed levels of historical endemicity [1, 2] are plausible when triangulated against other values reported from the pre-intervention era (for example, [3, 4]), but the relatively crude categorization of all-cause malaria endemicity strata and the cartographic approach used preclude a more formal quantification of the global *P. falciparum* endemicity declines and their link to urbanization beyond the broad relationships presented here and in the main manuscript. Nevertheless, recent mapping of the limits of *P. vivax* transmission (Additional file 6), indicate a very similar contraction as that seen for *P. falciparum*, and recent global spatial analyses have highlighted very similar impacts of urbanization on both *P.falciparum* [5] and *P.vivax* [6] malaria prevalence. Here we repeat analyses undertaken in the main paper to confirm that findings remain unchanged when switching to using these *P.vivax* limits to represent the contemporary situation. With contemporary *P. vivax* transmission being rarely more intense than mesoendemic [7, 8], historical endemicity levels likely correspond to *P.falciparum*, and thus we do not repeat the analyses described in section 4 here.

Unsurprisingly, figure 10.1 below shows very similar results to those found in figure 2 of the main document, with the vast majority of Americas and Asia countries showing greater rates of urbanization in those areas that became malaria free over the last century, compared to those that remained endemic, as defined by the contemporary limits of *P. vivax*. A mixed picture is again seen for the African countries. Figure 10.2 shows the same urban extent change plots as produced for *P.falciparum* in Additional file 9, but using *P.vivax* limits, and for countries where over 20% of the land area became malaria free, while over 20% remains endemic. Again, the same trend as for *P.falciparum* is seen, with increased urbanization in those areas where malaria was eliminated from, compared to those areas that have remained endemic.

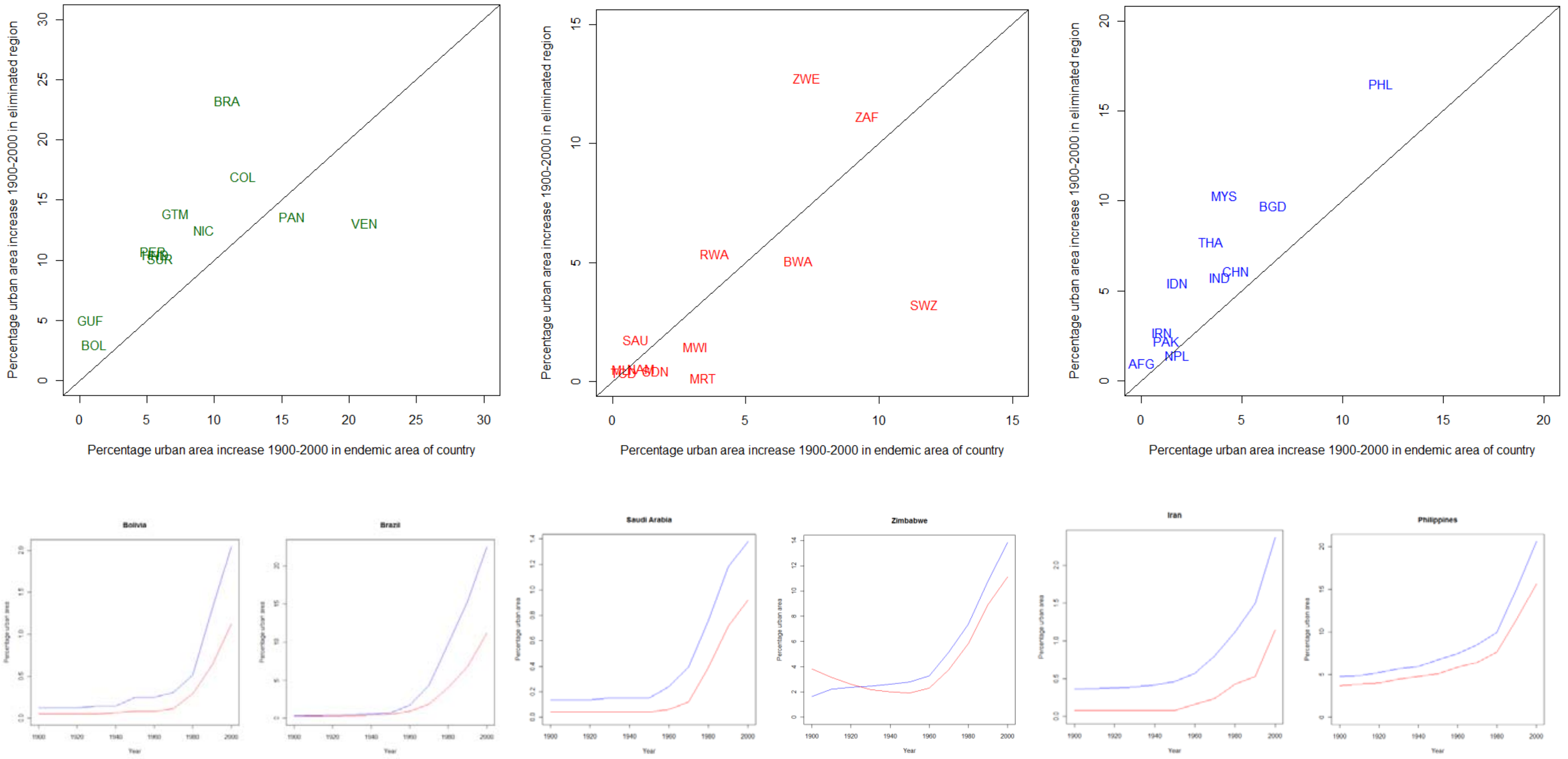

Figure 10.1. Plots showing urban area changes 1900-2000 between areas of countries that remain malaria endemic today (as defined by the limits of P.vivax transmission), and those that have undergone elimination for (a) the Americas, (b) Africa plus Arabian peninsula and (c) Asia. In each case, scatterplots of urban area increases in endemic versus eliminated areas with one-to-one lines overlaid are shown at the top, and example plots of trends in urban area percentages between areas that eliminated malaria (blue) and that remain endemic (red) are shown at the bottom (the full set of these plots is provided in supporting online material. The ISO country abbreviation for country name is used on the scatterplots (http://www.iso.org/iso/english_country_names_and_code_elements).

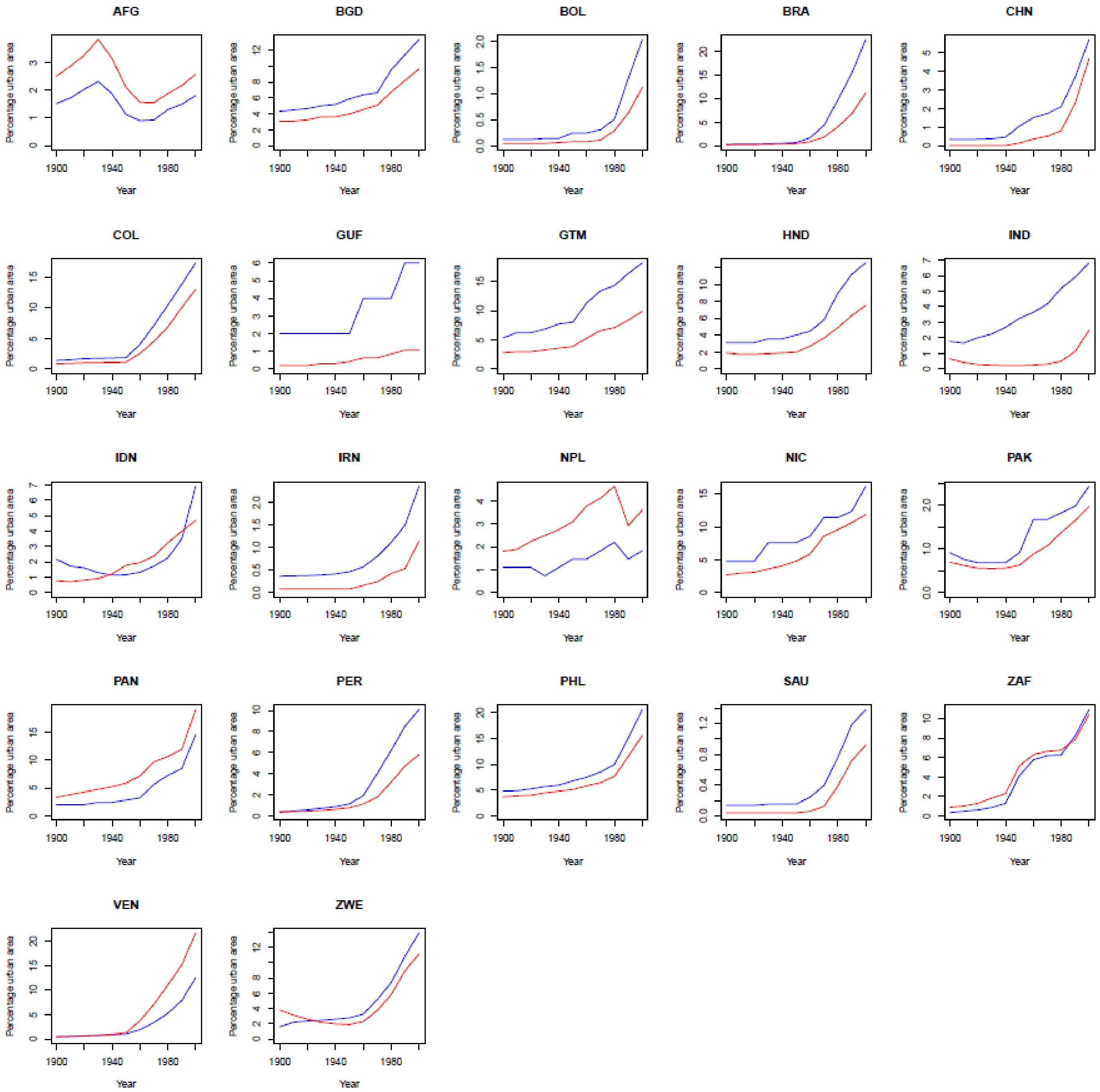

Figure 10.2. Plots of percentage of area that is classed as urban over time in (i) areas that underwent malaria elimination 1900-2007 (blue line) and (ii) areas that have remained endemic 1900-2007 (red line),as defined by the contemporary limits of P.vivax transmission, for countries where substantial areas have become malaria free between 1900 and 2007. See http://www.worldatlas.com/aatlas/ctycodes.htm for country codes.

References

1. Lysenko AJ, Beljaev AE: **An analysis of the geographical distribution of *Plasmodium ovale*.** *Bull W.H.O.* 1969, **40:**383-394.
2. Lysenko AJ, Semashko IN: **Geography of malaria. A medico-geographic profile of an ancient disease [in Russian].** Edited by Lebedew AW. Moscow: Academy of Sciences USSR; 1968: 25-146
3. Smith DL, Dushoff J, Snow RW, Hay SI: **The entomological innoculation rate and *Plasmodium falciparum* infection in African children.** *Nature* 2005, **438:**492-495.
4. Smith DL, Guerra CA, Snow RW, Hay SI: **Standardizing estimates of the *Plasmodium falciparum* parasite rate.** *Malar J* 2007, **6:**131.
5. Tatem AJ, Guerra CA, Kabaria CW, Noor AM, Hay SI: **Human population, urban settlement patterns and their impact on *Plasmodium falciparum* malaria endemicity**. *Malar J* 2008, 7:218.
6. Qi Q, Guerra CA, Moyes CL, Elyazar IRF, Gething PW, Hay SI, Tatem AJ: **The effects of urbanization on global *Plasmodium vivax* malaria transmission**, *Malar J* 2012, 11:403.
7. Baird JK: **Neglect of *Plasmodium vivax* malaria**. *Trends Parasitol* 2007, 23:533-539.
8. Gething PW, Elyazar IR, Moyes CL, Smith DL, Battle KE, Guerra CA, Patil AP, Tatem AJ, Howes RE, Myers MF, et al: **A long neglected world malaria map: Plasmodium vivax endemicity in 2010**. *PLoS Negl Trop Dis* 2012, 6:e1814.
