# Supplementary material for: Urbanization and the global malaria recession
Source: Malar J. 2013 Apr 17;12:133. doi: 10.1186/1475-2875-12-133 (PMC3639825; doi:10.1186/1475-2875-12-133)
Supplement: Additional file 11 — Urbanization and changes in malaria transmission. Description: Description and results of per-country statistical analyses of changes in malaria transmission intensity and urbanization levels. [file 1475-2875-12-133-S11.pdf]

**Urbanization and changes in malaria transmission**

The previous sections have highlighted the relationship that exists between urbanization and malaria elimination. Urban areas have been shown, in a contemporary context, to exhibit lower levels of transmission than surrounding rural ones [1], therefore, if the process of urbanization is a causal factor in malaria declines, a consistent pattern of greater transmission reduction in those areas that have undergone urbanization in the past century than those that have remained rural should be seen.

Figure 11.1 below shows the results of comparing the mean changes in transmission class from the historical to contemporary maps in areas that are urban today, versus those that have remained rural, for the endemic areas in 1900 of 158 countries. The plot shows that 82.3% of the countries showed a greater transmission reduction in their urban areas than rural ones. Of the 28 countries that displayed a greater malaria reduction in rural areas, half of these were in sub-Saharan Africa, where the smallest reductions in transmission occurred.

While these results confirm that generally, greater transmission reductions occurred in areas that are urban today, they do not indicate whether the largest declines in transmission were associated with greater *rates* of urbanization. We therefore examined whether the rate of urbanization was higher in regions of countries that saw the greatest reductions in *Pf*R0. Sixty-three countries were identified that had two or more *Pf*R0 reduction classes, each covering at least 20% of their land area, and the rates of urbanization 1900-2000 in the areas exhibiting the smallest reduction in transmission were compared to those in the areas displaying the greatest reduction in transmission. Figure 3 in the main document maps these results, and shows that 84.1% of countries included in the analyses had a greater urban increase in the areas that showed the greater *Pf*R0 reduction than those that showed the smallest transmission reductions.

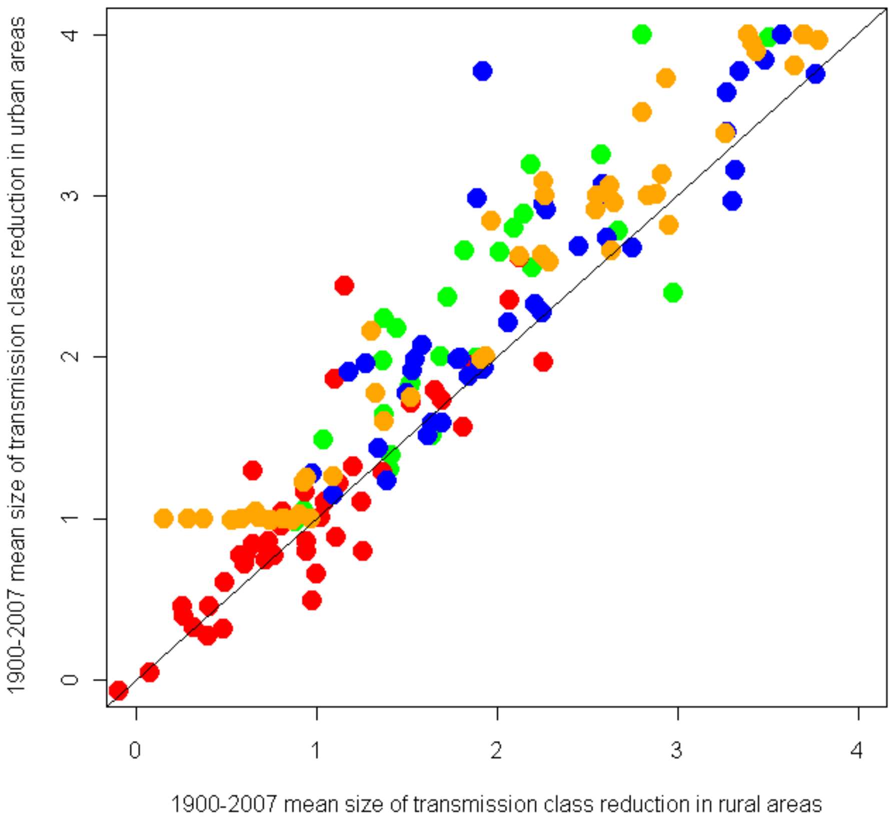


*Figure 11.1. Scatterplot showing the size of mean transmission class reduction in areas of countries that have remained rural over the past century, versus those that are urban today. Each dot represents a country and is coloured by region, where green = Americas, red = sub-Saharan Africa, blue = Asia and orange = North Africa and Europe. A one-to-one line is drawn to aid interpretation.*

**Reference**


1. Tatem AJ, Guerra CA, Kabaria CW, Noor AM, Hay SI: **Human population, urban settlement patterns and their impact on *Plasmodium falciparum* malaria endemicity.** *Malar J* 2008, **7:**218.
